# Cognition, Information Fields and Hologenomic Entanglement: Evolution in Light and Shadow

**DOI:** 10.3390/biology5020021

**Published:** 2016-05-21

**Authors:** William B. Miller

**Affiliations:** Independent Researcher, 6526 N. 59th St., Paradise Valley, AZ 85253, USA; wbmiller1@cox.net; Tel.: +1-602-463-5236; Fax: +1-480-794-1602

**Keywords:** hologenome, cognition, entanglement, quantum evolution, holobiont, zygotic unicell, self-organization, Darwinism, niche construction, information field

## Abstract

As the prime unification of Darwinism and genetics, the Modern Synthesis continues to epitomize mainstay evolutionary theory. Many decades after its formulation, its anchor assumptions remain fixed: conflict between macro organic organisms and selection at that level represent the near totality of any evolutionary narrative. However, intervening research has revealed a less easily appraised cellular and microbial focus for eukaryotic existence. It is now established that all multicellular eukaryotic organisms are holobionts representing complex collaborations between the co-aligned microbiome of each eukaryote and its innate cells into extensive mixed cellular ecologies. Each of these ecological constituents has demonstrated faculties consistent with basal cognition. Consequently, an alternative hologenomic entanglement model is proposed with cognition at its center and conceptualized as Pervasive Information Fields within a quantum framework. Evolutionary development can then be reconsidered as being continuously based upon communication between self-referential constituencies reiterated at every scope and scale. Immunological reactions support and reinforce self-recognition juxtaposed against external environmental stresses.

## 1. Introduction

The premise of this special issue is an enlarging perception that despite many appended forms, the Modern Evolutionary Synthesis does not represent a full account of eukaryotic evolution. For the last 150 years, as expanded into the Modern Synthesis and beyond, Darwinism has remained the unshakable center of standard evolutionary thought. More recent attempts at modification, such as the Extended Evolutionary Synthesis, still remain firmly anchored within the presumption of an obvious dominance of selection and random variation [1,2]. There is a tendency within that debate to wage deeply into details of theory, mechanism, nomenclature, and any perceived weaknesses or strengths of contemporary research. Yet, the issues as properly considered are actually few, and can be easily defined. Is evolution stochastic or not? If not random, is there a purpose? If there is a purpose, can it be considered a creative process at any scope or scale? Whether random or not, where is the central action of evolution, that is, where and what are its precise targets? Derivative to these primary issues are four further considerations. First among these is the one most vigorously debated: is natural selection ultimate causation, one factor among many, or mere tautology? Secondarily, is heredity primarily a vertical phenomenon or something other? This leads then to the third aspect so critical to the Modern Synthesis. Is evolutionary development best understood through the metric of gene frequencies or not? Lastly and little considered, how might eukaryotic organisms, as the endpoint of these processes, be best understood?

In order to gain a better perspective on these issues, it is fortunate that new research has revealed aspects of eukaryotic life that were not fathomed until quite recently. Eukaryotic life is holobionic by definition [3]. Its microbial fraction has only very recently been revealed through metagenomic sequencing and other advanced technologies and is much more elaborate and intimate than previously imagined [4]. Further yet, the complexities and extent of cell-cell communication that underpin cellular cognition have been generally recognized only in the last decades [5,6]. Lastly, there is now rapid progress in exploring the full extent of epigenetic impacts and their heritable transmission [7,8,9]. It is therefore contended that the impact of these relevant discoveries impels a thorough rethinking of evolutionary development with a decidedly changed focus.

As part of this shift, several concepts indicative of quantum systems can be included in the discussion. In particular, this includes the evaluation of biologic phenomena as existing in simultaneous states of ambiguous expression or probabilities of outcomes in genetic and cellular terms. In physical systems, this quantum duality is considered a superposition of probabilistic outcomes and chronologies in which a quantum state is considered a summation of two or more differing ones. A similar concept in biologic terms can be useful in understanding the deployment of epigenetic impacts and cellular responses to homeostatic stress [10,11]. Although these quantum phenomena properly dwell within the purview of quantum statistical mechanics and its rigorous use of statistical averages to define ensemble functions, general concepts based on quantum phenomena can still be deemed applicable. Such quantum principles include the inter-convertibility of physical thermodynamic principles into biologic action, quantum coherences that enable amplifying oscillatory phenomena in cellular activity, and non-local correlation through quantum entanglement (action at a distance). In each of these circumstances, biologic molecules and biologic expression do not necessarily exhibit one-to-one relationships and biologic molecules can entwine states at a distance without apparent direct connections [12].

Until recently, evolutionary theory has largely concentrated on the macro form. There has been relatively little attention to the unseen microscopic sphere or the immunological rules that govern it. The tendency has been to dwell upon the macroscopic aspect of our eukaryotic whole that dazzles in the light with much less scrutiny of the shadowed microscopic details.

In the Renaissance, great artists such as Caravaggio used compositional chiaroscuro, sharply drawn lights and countervailing areas of darkness, to create compelling images. Close inspection of any canvas of that type reveals that these master artists instinctively understood that the complexity of the shadows and their detail is as consequential to the whole as any portion that is vividly illuminated. The shadows are filled with texture, gradation, and variety. Indeed, if the shadows were withdrawn from that form of image, it would be rendered lifeless to our eyes. Our prior understanding of evolutionary processes has been based on rigorous examination of biological light with little emphasis upon two vital aspects of biology that deserve further inspection: the microbial fraction of eukaryotic life and the essential duality of information as both representation of physical actuality within context and a corresponding source of ambiguity. When both sides are fully honored, evolutionary biology can be productively reappraised in an entirely consistent and differing frame as a complex hologenomic entanglement, always predicated upon and faithfully rooted within cellular origins throughout eukaryotic evolutionary development [13].

## 2. Darwin, the Modern Synthesis, and Beyond

When Charles Darwin offered his seminal work, he was unaware of the existence of genes. Backed by scrupulous observation, he proposed that evolution proceeded by the gradual modification of heritable variations through a process of natural selection. Notably, Darwin was not the first to proffer the concept of natural selection, although he was its most capable advocate. The theory of natural selection had been advanced earlier by Patrick Matthew, a Scottish horticulturist, in 1831 [14] and Darwin was familiar with his work. It is also remarkable that the lively debates about evolutionary mechanisms of his time have a continuing familiar refrain. In particular, in the early nineteenth century, Lamarck’s proposal that individuals can acquire characteristics based upon the patterns of use of the various faculties had many advocates [15]. Even Darwin had countenanced a variant of Lamarckism that he termed “pangenesis” in his 1868 text, *Variation in Plants and Animals under Domestication* [16]. Indeed, the debate about the primacy of selection and whether evolution has direction or is random has been ever ongoing and vigorous.

In the later part of the 19th century extending into the early part of the 20th, a variation of Lamarckism, known as orthogenesis, was propounded by Wilhelm Haacke and then promoted by the German zoologist Theodore Eimer. They believed that an organism held towards a fixed course by internal forces. In their view, variation was not random and selection was not a powerful force since a species is carried forward automatically by inner dynamics [15].

The integration of genetics into Darwinism began in 1900 when Bateson translated an obscure paper by Mendel into English and began asserting its findings as fundamental to understanding heredity and evolution [17]. By the early 1920s the pioneering work of Fisher, Haldane, and Wright had developed into population genetics, the formal study of genetic variation and the distribution of gene frequencies under natural selection. A further critical contribution was Mayr’s identification of reproductive isolation as the cornerstone of speciation [18]. George Gaylord Simpson’s work then reconciled this new landscape with paleontology [15].

From those beginnings, through myriad contributors and continuing until today, the Modern Evolutionary Synthesis has itself evolved as the unification of Darwinian natural selection theory with the burgeoning science of genetics. Yet, its primary principles have remained stable: heritable variation is random and natural selection is the main evolutionary mechanism. Changes in genetic diversity and Mendelian segregation are best understood within the context of populations through vertical descent, and its occurrences are necessarily gradual [19,20].

In discussing the long journey from Darwinism to the Modern Synthesis, Massimo Pigliucci observes that when Darwin was writing his volumes, two major questions were considered paramount; how can the diversity of life and its history be explained, and then further, how might that account for the apparent match between form and function in organisms [21]? Prior to any explicit knowledge of genetics, evolution theory was, in its earliest stages, a theory of forms. In contrast, during more recent decades, evolutionary theory has become nearly exclusively a theory of genes. Much of this perspective is due to the seminal work of Haldane, Fisher, Dobzhansky, Sewall Wright, and Mayr in exploring statistical methods within population genetics. Each was attempting to account for variation through the integration of Mendelian genetics into both the micro and macro evolutionary landscapes [22]. Of major concern was whether the microevolutionary changes that could be cataloged in local populations might be explicitly reconciled with the novelty and morphological inventions seen within macro evolutionary trends, and yet, remain in allegiance to a presumption of the primacy of natural selection that exerts its force over geologic intervals [23]. The neutral theory of molecular evolution proposing genetic drift as a major evolutionary driver was a consequential revision. Most mutations were envisioned as selectively neutral and not directly affecting the fitness of organisms [24]. Therefore, the understanding of selection shifted. It was not just a positive action, but instead, a purifying one through the elimination of the most harmful mutations with fitness accumulating by drift [25,26].

Over time, a trend emerged to accommodate a more pluralistic narrative compared to the major tenets of the Modern Synthesis [27]. Although not the first to champion it, Margulis [28,29] became a vigorous proponent of incorporating symbiogensis and endosymbiosis into the evolutionary narrative by outlining an organelle genesis theory [30]. McClintock played a similar crucial role with her illuminating work on transposable elements [31]. In particular, the discovery of the homeotic *Hox* master genes in the 1980s, highly conserved across many phyletic divisions and over a vast continuum, has gradually altered the focus of research towards regulatory complexity compared to the composition of genes [32,33].

Others researchers have stressed aspects felt to be essential parts of genetic evolvability though still maintaining coherence with overarching natural selection. Radman *et al*. stressed genomic variation, recombination, and mutation [34]. Caporale reviewed molecular biological mechanisms within a pleiotropic genome that responds to stress in a non-random and strategic manner [35,36]. Fodor and Piattelli-Palmarini in 2010 offered that natural selection in and of itself cannot explain evolution and emphasized what they appraise as extraordinary creativity in genetic evolution [37]. Jablonka, Lamb, and colleagues have reconsidered the Modern Synthesis by concentrating on Lamarckian epigenetic factors, suggesting environmental impacts are of major importance beyond intrinsic genes and random variation [8,38,39]. Others, such as McFadden [40] and Ho [12] have attempted to reconcile evolution with the natural sciences and quantum physics.

As part of the crosscurrents of thought, and starting in the 1960s, the Williams revolution shifted the frame of reference from population genetics towards models of natural selection through kin selection. The focus became the gene as a fundamental unit of self-preservation, accounting for both fitness and altruistic behavior through the inclusive fitness of a larger gene pool. This concept was further expanded by Dawkins [41], Doolittle and Sapienza [42], and Orgel and Crick [43]. It was maintained that genomic expansion was largely due to the repeats of selfish elements within a genome thereby accounting for “junk” DNA.

In 2007, Rose and Oakley offered an extensive critique of the Modern Synthesis. Certain aspects were no longer tenable, such as viewing the genome as a “well-organized library of genes” [27] that have single functions shaped by natural selection. They offered a greater emphasis on horizontal gene transfer, gene duplication, symbiogenesis, and differential lineage assortment. In a review of evolution from the perspective of new findings in genomics in 2009, Koonin contended that these studies indicate that natural selection is not the only force that shapes evolution and may not even be dominant. Non-adaptive forces might be the greater fraction. Even further, Koonin suggests that there is “hope for the discovery of simple ‘law-like’ regularities” [20] that underpin evolutionary development.

Others have distanced themselves from the Modern Synthesis to an even greater extent. Woese and Goldenfeld in 2009 urged casting away the Modern Evolutionary Synthesis to permit a fuller reconsideration of the last century of dogma in favor of a full integration of evolutionary theory with microbiology and molecular biology [44]. Shapiro has provided a link towards that goal, calling for a critical rethinking of evolution with natural genetic engineering rather than natural selection as the major mechanism [19].

Perhaps the most comprehensive attempt at a full and comprehensive alternative to the Modern Synthesis has been the promulgation of an Extended Evolutionary Synthesis (EES) [1,2]. This represents a pluralistic approach that views the center of action in evolution as developmental or phenotypic plasticity enabling an organism to change its phenotype in response to the environment. In this frame, heredity extends beyond genes to encompass the heritable transmission of other developmental resources between parent and offspring that can be both bioactive and behavioral in nature. Significantly, such effects are not merely confined to germ to germline transmission, but can also extend soma to germ, or germ to soma. Through these mechanisms, there is a tendency towards mutual reinforcement through niche construction, *i.e.*, reciprocal causation between the capacities of the organism and the outward environment with each impacting the other. In this manner, an organism can shape its own developmental trajectory by adjusting both internal and external states along constructive developmental paths. Therefore, adaptations arise by both natural selection and a separable process of internal and external constructive development in reaction to epiphenomena.

Yet, EES still represents another pluralistic adjustment to the Modern Synthesis rather than any revolution. The targets of selection are changed and limits are imposed, but the underlying narrative receives no definitive challenge. By what means might evolutionary theory be fully reconsidered so that natural selection is no longer its centerpiece? The requirements would include a complete reappraisal of the targets of natural selection and then, even more importantly, a fundamental change in the means by which biological organisms are construed. Further too, it would require an ecobiological construct that is not merely a direct reduction to allele frequencies [45].

## 3. Cognition is Fundamental

In effecting any disassociation from the standard evolutionary narrative, contemporary resources from the emerging fields of hologenomics, metagenomics, and epigenomics can be productively applied. However, as important as those disciplines are in any attempt to suggest a new synthesis in apposition to Neodarwinism, one decided advantage is the opportunity to begin where Darwin could not. That differentiated platform is the centrality of cognition to life [19,23,46,47]. In 2011, James Shapiro stated it plainly, “Life requires cognition at all levels.” [19]. Beyond metaphysical speculations, Darwin did not have any concept of its biological ubiquity nor did any of the theorists of the early through mid-20th Century. Yet, even when the last few decades of research have revealed that self-referential cognition underscores all life on the planet [48,49], and further, that it might be productively considered and dissected apart from metaphysics, it has attracted the interest of few evolutionary biologists.

In 2007, Shapiro wrote, “Forty years’ experience as a bacterial geneticist has taught me that bacteria possess many cognitive, computational and evolutionary capabilities unimaginable in the first six decades of the twentieth century.” [50]. That assertion is based upon the extraordinary range of metabolic cellular processes exhibited by bacteria and used to evaluate and monitor their own internal environment. It can thereby be advanced that each living entity accomplishes these activities for the maintenance of self-identity, and further, that these actions are reinforced through willing cooperation. It is now well established that the engagement of bacteria in the colonial form results from abundant multicellular collaborations under girded by sophisticated mechanisms of intracellular and cell-cell communication [51,52]. As Lyon [47] observes, bacteria have an extensive cognitive toolkit that includes a wide range of faculties: advanced sensing, communication, autoinduction via the indirect use of information gathered by proxies, some elements of sociality, various forms of motility including complex swarming behaviors, and memory. Given the variety and sophistication of these actions, there is specific evidence of some elemental level of cognitive function at every scope and scale applied towards the maintenance of self-awareness that, in turn, permits such levels of collective sensing, cooperation, and interdependence. All these functions require levels of memory and information processing and are positively directed towards problem solving [53].

Ample complex cooperative strategies are clearly demonstrated throughout diverse eukaryotic cellular ecologies. The human gut and other tissue sites demonstrate that the depth of those interrelationships is great enough to promote specialization in the production and use of resources [4]. Therefore, microbes and individual cells send, receive and interpret information and importantly, put such outputs to use according to their scale to enact and maintain both individual and collective homeostatic preferences. They do so not merely based on their own immediate and explicit environment but based upon cues that are responsive to more global concerns that emanate from entire cellular networks [54]. The level of sophistication of these communication and feedback mechanisms provides an instructive comparison with our own human economic framework [55]. As such, economic equilibrium theory has been applied to the cellular biotic realm based upon bacterial metabolic exchange vis-à-vis the trading of resources to create a general equilibrium model that is useful for understanding both bacterial and human reactions. It is, therefore, implicit that widely disseminated intercellular processes lie at the center of a complex chain of cooperative cellular behaviors that characterize the biosphere and are then reiterated at every scope and scale to even include our own human proclivities. This discrete interaction helps explain why auxotrophs, or highly specialized cells unable to produce essential metabolites, are prevalent in symbiotic and free-living bacteria and appear to drive biosynthetic gene depletion as a fitness adaptation [56]. In sum, they have staked themselves upon trading for resources and an existence through cooperation and the exchange of information. That such cells exist indicates an expectation of entangling reciprocity as an inherent biological reality and then further, underscores an underlying biologic imperative for cooperative action as a centerpiece of biological activity. It can, therefore, be asserted that cooperation is the conditional basis for the construction of new levels of organization implicit to all evolutionary development [57]. Cooperative interchange exists throughout biology, whether at the level of individual cells or eukaryotic multicellular organisms. Mutual reciprocation between biological entities and the external environment is omnipresent [58]. These expectations and dependent phenomenon are so commonplace that it exists beyond communal circumstances and is also known to be evident among free living bacterial cells [56].

Such interactive behaviors are not exclusive to the unicellular side of the microbial sphere. It is now widely acknowledged that viruses are an essential element of our evolutionary narrative [20,59,60]. All of the critical functions of cells such as replication, translation, and repair are of viral origin. Our genome has thousands of endogenous retroviral sequences [61] and it has been a more recent surprise to identify that there are also large numbers of viral sequences that have impacted eukaryotic evolution [62]. The impact of the virome extends well beyond pathogen and host interactions and extends into every aspect of eukaryotic life to such depth that it is has been proposed that this component might determine our “normal” transcriptional state [63]. Further, it is clear that viruses and sub-viral particles exhibit a range of intelligent behaviors. They are efficient problem––solving entities, capable of overcoming the most sophisticated cellular mechanisms. They can evade or change cellular immune systems to meet their requirements and participate in and control the transmission of information between other biological entities [64]. Viruses cooperate with each other to determine cell fates [65], and there is complex communication and exchange of information between phage and bacterium that determines survival, reproduction, and movement [66]. Their actions as bacteriophages require sophisticated highly coordinated mechanisms for entering cells requiring the recognition of a wide range of bioactive molecules [67,68]. Therefore, it is clear that communication between all microorganisms is widely distributed and abundant [69] to such an extent that Visick and Fuqua liken its pervasiveness to “chatter” [6].

There is no doubt that all microbes including bacteria, viruses, and even prions have discriminatory preferential states. It is the reliable partialities of specific microbes for certain tissue types that form the definable criteria of infectious disease dynamics upon which the clinical practice of medicine is based [23]. Lyon has queried whether extensive signaling transduction pathways that have been demonstrated in microbes form a coherent adaptative response [47]. Direct observation asserts that microbial responses are indeed predictable and reliable in many instances. Lyons offers this, “Biological cognition is the complex of sensory and other information-processing mechanisms an organism has for becoming familiar with, valuing, and [interacting with] its environment in order to meet existential goals, the most basic of which are survival, (growth or thriving), and reproduction.” [47] However, those capacities are not exclusive to bacteria, or viruses, but have been shown to exist within all living entities including the individual cells of any eukaryote as they experience stress and make individual coping decisions [70]. Importantly, therefore, all biological mechanisms such as physiological traits underscore abilities that are best understood as direct exaptations of the unicellular state [71,72].

If it is then granted that cognition is a consistent element of microbial and cellular life, how might such a faculty have arisen? Since cells and microbes are entities that have some form of awareness of condition and are bounded compartments, it might be considered that in order for any awareness of condition to supervene, boundaries must exist. Obviously, without such perimeters, there is only one continuous state. Hence, borders are crucial for awareness and it might be surmised that it arises as a phenomenon of coherence induced by the bounded state in which physical forces are entrained, perhaps as a special case thermodynamic quantum coherence [73]. In that regard, there is research that supports that quantum processes are essential to life [74,75]. Such activity appears to be demonstrable within eukaryotic cells. In particular, the intracellular components of the cytoskeleton appear to be dependent upon quantum phenomena. Microtubules demonstrate coordinated vibrational beat frequencies that may produce quantum coherences [76]. Tubulin, actin filaments, collagen, non-polar protein interiors or membrane lipid peroxidation processes interact with the vibratory capacities of microtubules either directly or via serotonin production promoting quantum signaling that permits the collapse of the superimposition of possibilities inherent to quantum phenomena [77,78,79,80].

Therefore, it can be surmised that awareness is both knowing something has entered its space and the awareness of it as information that can be channeled through quantum inferences that devolve towards the physical realm and can then be used to resolve cognitive ambiguities [81]. Coherence actualizes the ability to discriminate preference within a frame that might otherwise remain ambiguous. Under such conditions, cognition is then the ability to purposely attempt to resolve ambiguity and, at a higher level, becomes the faculty of maintaining higher levels of ambiguity prior to initiating action even if resolutions can be sensed.

Yet, an awareness of condition or any self-referential capacity can be separated from other aspects of intelligence. One person may be better at solving certain problems than another, but our assessment of intelligence is not enlarged to presume that any basal sense of “self” of one individual is greater than another. It is, therefore, possible to consider “self” as separable from other aspects of cognitive discrimination and ability. It is can then be asserted that bacterial and cellular self-awareness exists as a condition of life but is still distinguishable from overall intelligence. Yet, bacteria are far from simple. As Shapiro points out, “The first point is to recognize that bacteria are far more sophisticated than human beings at controlling complex operations.” [50]. Bacteria use chemotaxis to find nutrients, avoid toxic chemicals, sense pH, and extensively interact with others. Therefore, the origins of cognition must originate in the physical world as an impulse that transmutes into biological form and is capable of refinement dependent upon scope and scale. However, self-awareness is better understood as a condition of life as a first principle upon which all resultant life rests.

In that regard, De Loof [82,83,84] has suggested that life should not be considered a noun, but a verb. In those terms, life must be regarded as the sum total of all executed acts of communication at any moment, at all levels of any compartmental organization, and as a summation of all that activity. Furthermore, all of that life activity is directed towards problem-solving. De Loof asserts that communication/problem-solving precedes selection and should therefore be considered a universal element of evolution.

Proceeding within the context of life as a verb, it can, therefore, be represented that life consists of the active use of information to sustain change towards preferential conditions for any living entity. In this regard, De Loof maintains that communication is the handling of information in a system that is organized as a “sender-receiver communicating compartment”. Yet, information is not merely data to any receiving entity. Upon being decoded by any receiver, it becomes part of the stored energy within the receiver that can be mobilized towards action as work. Therefore, for De Loof, the cell concept should be changed from the strictly material towards a larger consideration of the cell as a “sender-receiver” universal unit of structure and function of all living matter from which complexity then builds from level to level.

In such a system, a natural bridge exists between thermodynamic considerations and a biological one that is best appreciated through quantum phenomena in which energy and information are considered essentially equivalent stipulations that are dependent upon receiver status in biological contexts. Although it is typical to consider biology in terms of organization in violation of the 2nd Law of Thermodynamics, the flow of information that is inherent to life processes with all the ambiguities that it creates is not usually part of that account. Considerations of entropy in living systems are by no means direct. For example, although it is common to consider information as concrete, communication is otherwise, generally pervasive, and often not directed towards any specific receiver. Instead, it is a generalized attribute of cognitive life and largely noise. Since the sending of information requires the release of energy, the amount of entropy in a system consistently transitions and is dependent upon the reception of information that is used to resolve ambiguities *versus* the quantity that remains noise.

Jacob *et al.* examined Schrödinger’s ideas about the fundamental requirements for life from the perspective of contemporary observations about bacterial self-organization and the emerging understanding of gene network regulation mechanisms and dynamics [85]. Schrödinger proposed that consumption of negative entropy requires the further context of an organism’s ability to extract latent information embedded within any environment from its complexity [86]. By acting together, bacteria efficiently perform this task through cooperative behavior and thereby prove their biotic cognitive ability and that of any basic cellular unit. When viewed in this manner, there are then direct links between thermodynamics and self-referential cognition. Even though biological organisms can be considered secondary to entrained thermodynamics, energy is merely in transit despite any temporary storage for work. Therefore, it is appropriate to consider all biologic organisms, either at the unicellular or multicellular levels as transient intermediary manifestations of energy flux in which information is part of that same phenomenon. In such a context, energy as information may be best framed as a phase transition in the physical order, such as water to steam. In our biological system, such a phase transition has been validated experimentally through calculations of the probability of a fluctuating neuronal membrane voltage exceeding certain activation thresholds that define neural coding [87].

A similar phase transition has been previously applied to the origin of life which has been likened to a physical transition such that information is transformed to achieve context-dependent causal efficacy over the matter from which it was instantiated [88]. Even though biological organisms can be considered secondary to entrained thermodynamics, energy is merely in transit. Contemplating such a transition requires our distancing ourselves from the manner in which we have traditionally considered any organism and accept the same dynamical frame of Walker and Davies [89]. In this circumstance, living organisms are way stations of entropic and enthalpic flux in which information as energy gains efficacy over matter. Further, since any such entity radiates energy via heat, it is then also continually effecting information transfer insofar as it is always radiating heat or other by-products of life processes. Among living things, this is ever ongoing and includes metabolic products as waste, cast off cells and particulate matter that have never traditionally been considered information but decidedly are. In this manner, all living things are dynamical agencies contributing to entropic flux on a steady basis towards the large universal entropic sink as entities that dissipate heat and information. In quantum circumstances, all such activities proceed from the superimposition of possibilities that is inherent in the biological sphere [90].

Therefore, any living organism is a temporary non-equilibrium dissipative entity a fleeting manifestation of the collapse of the superimposition of possibilities of a variety of entropic and enthalpic moments as rates of change that are reflective of homeostatic status. A number of variables determine that state: temperature, volume, pressure as well as entropy and enthalpy. Each are state functions and cellular life and homeostatic status depends upon them. Such variables may be difficult to measure, but there are natural bridges between thermodynamic exchanges and biologic entities that employ all of these factors. Photosynthesis for the direct conversion of energy from sunlight to sugars required for metabolism and growth by phototrophs is an obvious instance. Chemotrophs extract energy for the manufacture of sugars by taking electrons from substances in their surrounding environments—a process called chemosynthesis. Other biologic entities, chemolithoautotrophs, get their energy from the oxidation of inorganic substances. *Shewanella loihica* PV-4, a metal reducing bacterium, can self-organize as an electrically conductive network becoming a long distance electron transfer conduit using outer membrane proteins and semiconductive minerals [89]. Instead of energy from the sun, or the inorganic molecules from deep sea thermal vents, these bacteria seem to represent a third different type of energy ecosystem in which microbial activity is sustained by the direct use of electrons available in the environment [91].

Such bioenergetic solutions can be productively regarded as fundamental principles of physics channeled into biologic expression. It would seem reasonable assume then that any system of cognition is energy dependent, and that energy flows via those quantum processes that represent that particular union along a continuum of physics as biology expression. The cusp can thereby be considered an inherent duality incorporating both the exchange of information and the reciprocal transfer of energy between receptive entities. Consequently, this can be properly represented as a first- order entanglement between the physical realm and the biologic one. A further supposition would suggest that cognitive self-awareness, as a quantum state, arises as a phenomenon of coherence induced by any bounded state in which the appropriate resonant energy is entrained. Within the eddies and flows of the varied gradients within the cell, or even within a viral capsule, when “life” supervenes within cohering physical boundaries, the resonant energy of awareness simply exists. Furthermore, then, as Whitehead conceptualized, it might not necessarily be invested only in bioenergetic molecules [92]. Whatever the reality, it is enough that it is evident in all that are regarded as “living”. However, as energy transfer is an oscillating function of frequency and amplitude, there must be zones of coherence (amplification or resonance) or decoherence that occur across gradients within any boundary condition. In biological terms, this can be considered regions of more or less ambiguity in which the superimposition of possibilities is either broader or more constrained. These can be considered as points of intersection of energy/information transfer within the cell as they overlap energetic inputs emanating from outside cellular margins. Within the cell, such zones of coherence can be regarded as foci of discrimination and preference enacted in biologic form as cognition, perhaps centered within cellular microtubules as has been suggested by Hameroff and Penrose [76]. Since information is energy transfer, information becomes a gradient function subject to harmonics and resonances that instantiates or promotes a spectrum of awareness of status. Therefore, each cellular unit is a coherent and discrete cognitive entity in which information becomes another form of resonant energy both within and without the cell. Energy becomes information within the bounded resonating chamber of the cell achieving the coherence necessary to become information to both sender and receiver. The difference between energy and information can then be assumed to be based on specific energetic coherences that permit its use by an apt receiver to settle specific biological ambiguities.

Life is best defined as the property of self-awareness that permits the use of information to either sustain or change conditions. Further, life as self-awareness is thereby imbued within everything that is regarded as living. It necessarily follows then that self-awareness exists independently of the number of steps required to enact it. Therefore, self-awareness is properly considered a state function. From that inherent base, its variability exists as a reiterative conditional function based upon discriminated preferences within varying frames of ambiguity. Under such circumstances, cognition can, therefore, be understood as the ability to purposely attempt to resolve ambiguity through the use of information. As a derivative then, at reiterative levels beyond the unicellular domain, cognition can be considered as the self-organizational ability to permit higher levels of ambiguity prior to the initiation of action, even if earlier resolutions can be sensed. Certainly, it must continuously be based upon basic thermodynamic principles of energy utilization and information transfer. Under such circumstances, however, free energy in thermodynamic terms becomes uncoupled from variational minimal free energy in biologic information space [93]. Others have upheld the statistical power of Markov blankets. In a Bayesian network, a Markov blanket is a set of nodes that consist of parents, its children, and any other parents of its children in which the probability distribution of each node is conditionally independent of the other nodes in the network. A set of such nodes of diverse parentage that connects to neighboring nodes can be considered a pertinent depiction of the means by which cellular membranes uphold their intracellular matrix as opposed to extracellular influences [94]. In those systems, inputs are based on Bayesian inferences of random inputs and typically too, local coupling [95]. However, within a context of self-referential cognition, there are direct biological limits placed upon the bounded dispersion of sensed states by which cells experience epiphenomena and the outward environment. Any inevitability of self-organization as a form of active Bayesian inference is thereby empowered as the means by which biological uncertainties are resolved through inputs that are not necessarily random and also through quantum biologic phenomena that are subject to both local coupling and non-local correlations [96]. Therefore, higher levels of intelligence can be understood as permitting an organism to resist the collapse of the superimposition of possibilities to improve decision-making within its environment. Intelligence is, therefore, discrete problem-solving that is context dependent, but is still separable from self-awareness.

If all living units are considered sender/receiver units, then all organic systems register information and further yet, transform it as part of inherent information systems. Intelligence as problem-solving beyond self-awareness might then properly be considered as an emergent property of an information system [97]. Further yet, intelligence should be more fully considered as the purposeful use of information which has been argued exists even at the level of a small protein [98]. Even so, intelligence is difficult to localize within any one structure of any macroorganism and might be better understood as an emergent property of any living entity as a part of a cellular network that both sends, receives, and interprets information. As Pookottil notes, jellyfish have no brain, but are self-aware and intelligent, demonstrating wide-ranging behaviors and advanced problem-solving abilities [97].

Therefore, it is best to consider intelligence as problem solving that has additive and emergent properties that extend from self-awareness but is also separable from it. Such capacities are much more widely present than previously understood. For example, Shomrat and Levin demonstrated that Planarian flatworms are able to reiterate their entire body including their brain if segmented, and will still demonstrate some intact memories from the initial brain structure [99].

Most discussions of intelligence have concentrated on an in-depth examination of animal behavior. Yet, plants have been considered nearly passive and their cognitive abilities have received little attention. However, they have memory and intelligence, and clearly demonstrate cognitive awareness through solving problems such as optimal light acclimation, transpiration, and resisting immunological transgressions [100]. Plants are capable of learning complex signaling behaviors, acquire large amounts of information and have the capacity to memorize and organize learned responses [101]. For example, *Mimosa pudica* exhibits clear habituation, suggesting some elementary form of learning. Unexpectedly, *Mimosa* can display this learned response for more than a month between stimuli. This relatively long-lasting learned behavioral change as a result of previous experience matches the persistence of habituation effects observed in many animals.

Nor have we understood the varieties of intelligence or its distribution. Cephalopod intelligence seems unlike our own. Cephalopods such as squid, demonstrate a form of highly distributed intelligence with independent motor control distributed to each of their arms and a system of highly sensitive chromatophores in their skin [102]. These cells demonstrate activity that is independent of the central nervous system and can be considered separate cognitive centers [103].

Therefore, cognition can be regarded as the purposive use of information and communication as represented across the entire microbial sphere and widely distributed in different cellular ecologies within multicellular eukaryotic organisms. Naturally then, it exists beyond any centralized brain structure. It is important to emphasize that the purpose of cognition is not merely reaction to stress. In biological organisms, information is also used for prediction that can also be understood in the context of resolving biological ambiguities towards biological expression. Such predictive capacity is universally distributed and is clearly exhibited by bacteria in which it has been demonstrated as a form of associative learning that has typically been attributed only to metazoan nervous systems [104,105].

With these considerations, an assertion can be made that self-awareness is a condition of life as a state function derivative from physical processes. Consciousness as awareness of an external environment is conditional to all forms of life and represents the specific differentiating junction between the biotic and abiotic realms [106,107]. In that frame, self-awareness is the deployment of information as another form of energy. Since life is self-awareness by definition, and further yet, since information is a form of energy transfer purposed towards settling biological ambiguities, then life, and then too, self-awareness, are properly regarded as a specialized form of energy transfer. The preservation of self-awareness is then best considered as a quantum process. Derivatively then, the subjective assessment of “self” becomes fundamentally related to the status of the participant/observer relative to others [108]. Under such circumstances, as Fingelkurts *et al.* assert, consciousness as we experience it becomes a neural collective phenomenon dependent upon a “nested hierarchy of electromagnetic fields of brain activity” in which subjective and objective reality represents a “unified metastable continuum guided by the universal laws of the physical world such as criticality, self-organization and emergence.” [109]. Therefore, within this quantum framework, it can be advanced that “self” is a quantum phenomenon experienced through the continuous collapse of the superimposition of possibilities that constitute the resolution of biological ambiguities inherent to the manner in which biologic organisms obtain information.

Therefore, a new beginning permits distancing from Darwinism as a precondition of any further evolutionary narrative. Self-referential awareness as a state function from the inception of life forward has its base value independent of the history of the system. Its broader expression as intelligence implies a wider range of problem-solving tools and remains an emergent phenomenon that is context dependent and causally related to its historical path. Self-awareness must arise from the physical system that preceded it and is thereby best understood as a function of entrained thermodynamics. It is likely then that awareness is based upon energetic coherences enacted primarily within the requirement of cellular boundaries including membranes or viral envelopes that permit conditions for any phase transition by which energy becomes information.

Since eukaryotes, bacteria, viruses and virions, and even prions are separable from inanimate entities through a reactive awareness of status, they too exhibit a property of self that is “life” by definition. Therefore, all known life and its consequent evolutionary course should properly be considered as based upon self-referential awareness, dependent upon contextual energy transfer as both information and communication with its attendant layers of uncertainty. Self unites with thermodynamics through these quantum uncertainties as the continuous resolution of ambiguities into biological expression. Furthermore, since the purpose of information transfer and communication is problem-solving and further yet, that this impulse originates with cellular mechanisms, it can be expected that evolutionary development would remain faithful to cellular imperatives throughout its course [71,72]. Specific evidence for this is available, as shown in the recent elucidation of the structure of the ribosome from its origination 3.8 billion years ago, with layers of accreted complexity as terminal additions extending forward continuously from an initiating central core [110].

## 4. A Differing Endpoint

At least as important as any new point of origination may be to any reconsideration of the Modern Synthesis, a fuller understanding of evolution is necessarily dependent upon an accurate perception of the current endpoint of these processes. In this regard, the general Darwinian appraisal of macroorganisms as unitary beings is no longer contemporary. No accurate current assessment of evolution can be undertaken without a thorough appreciation of the essential nature of all eukaryotic organisms as holobionts. There are currently estimated to be at least 100 trillion microbes that are in and on us—bacteria, viruses, fungi and others. They outnumber our primary cells by a factor of 10 to one or more [111]. Further yet, if the entire genetic fraction of any holobiont were to be considered, then the full genetic cohort of the associated microbiome outnumbers our innate genetic complement by 100 to one or more [112]. Although there has been a movement toward revision of the raw numbers [113], the conclusions about the nature of eukaryotic multicellular organisms as functional holobionts remains steady.

Research is now underway to properly define our dependencies upon our microbial partners for the proper function of our gut [114], brain, and central nervous system [115,116] and immunesystems [117]. In view of these interrelationships, Gilbert *et al.* have discussed considering all eukaryotes as multi-species units [118]. However, any complete understanding of evolution requires a complete separation from our prior subjective notions. Indeed, the entire model of “host” and “guest” should be revised. Rather than regarding any macroorganism as an inherent singularity, a more accurate comprehension restates eukaryotes as vast collaborative enterprises of co-linked, cooperative, co-dependent and competitive ecologies merged together so seamlessly as to seem one discrete entity [23]. All multicellular eukaryotes are holobionts. There are no exceptions and its implications must be considered in any appraisal of evolutionary development.

The concept of the hologenome has been championed by Eugene Rosenberg and Ilana Zilber-Rosenberg [119,120], although it was originally advanced by Richard Jefferson years earlier [121]. The hologenome theory of Rosenberg and Zilber-Rosenberg maintains that the actual object of natural selection extends beyond any macroorganism as “host”. Instead, it extends to encompass the entire symbiotic community with which it is associated. However, within their theory, the traditional concepts of “host” and “guest” are strongly maintained even as they consider this duality as a conjoined unit of selection. Furthermore, their evolutionary narrative remains an entirely traditional Darwinian one. Conceptually then, their approach is not specifically different from the Synergism theories of Maynard Smith and Szathmáry in which the object of selection is the synthesis of collaborative components at many levels and at major transitions [122]. With these theories, the object of selection is shifted by an enlarged pluralistic bandwidth beyond the central genome of a macro-organism but remains centered within selection theory.

Chiu and Gilbert regard multicellular eukaryotes as holobionts with multiple species of persistent symbionts [123]. Whereas they do appreciate the anatomical, physiological, developmental and immunological unity of holobionts, their interpretation is that it is best understood as an instance of “reciprocal scaffolding” in which species share relationships. Therefore, symbionts are more than mere appendages and are part of a “superorganism”. However, Miller asserts that the intimacy of the relationship is intimate enough that holobionts are beyond reciprocal scaffolds, or even superorganisms, but are instead better understood as assemblages of linked cellular and viral ecologies as distinct merged confederacies into a unique complex integrated entirety [23].

Therefore, the combination of eukaryotic “us” and “other” must be reappraised within a consensual “we”. In this manner, macroorganisms are no longer evolutionary singularities but are always the product of the mutually collaborative and competitive needs of conjoining cellular action in a transient arc of life that to our casual human appraisal is “personhood”. Such oneness is merely seamless integration. Necessarily, such consensual links require the backdrop of two inter-related features of cellular life: Information sharing among the variety of confederated mixed cellular ecologies that must be constrained within immunological rules foundational to the maintenance of mutual co-alignment.

When multicellular eukaryotes are reconsidered as always anchored within cellular mechanisms that extend across many mutually co-linked life forms, information systems and information transfer become the logical framework for any deeper understanding. In 2002, Lloyd introduced the concept of the Pervasive Information Field (PIF) in order to attempt to define a system of self-organization that is universal and scale free upon which many inter-related disciplines could be based [124]. Such an information field offers insight into information storage and its usable and accessible distribution and has been used as a model for the description and modeling of social systems [125]. Clearly, life is a unique type of information management system that is distinguished in character from theoretical measures such as Shannon information. The difference centers on context as apart from raw data [88]. It is certain that information is being sent and received within and across the cell at all times, reverberates externally and has further reciprocal effects. The context of information transfer across a vast multicellular constituency is obviously complex and based upon receiver and sender characteristics which is perforce a function of velocity that depends upon the medium of transfer and information type. Further yet, a great deal of it might be regarded as noise. The appropriate means of assessing its summation might be best considered as a complex information field, and in turn, such an active informational field has its epicenter within and overlaps every cell and projects beyond it. This is simply analogous to the more familiar concept of any cell having its own energy field that consists of its gradients and fringe effects. In the case of an information field, it is the summation of all the sources and receptors of information within the cell and extends outward into the external environment. In this regard, the concept is similar to the summation of communication approach of De Loof [84].

The term “field” is appropriate since there is no reason to suspect that there is any exclusivity for reception of information within any purported “network”. Some players might be privileged based on field effects, e.g., amplitude or frequency, but it is likely an open system, more like a broadcast than a direct line. When a virus enters a cell, it is able to tap into the information field and utilizes it to begin its intracellular purposes. Furthermore, since information has velocity and degrades over distances, then it is a gradient phenomenon with fringe effects and distortions. This becomes a primary source of ambiguity within biologic systems substantiating the contention that life can only be understood as the continuous resolution of uncertainties within context.

Any concept of a Pervasive Information Field can be easily reconciled with self-awareness. It is an actualization in biologic terms of an informational set. Within this definition, it rationalizes the non-intuitive requirement of cellular boundaries towards purposive self-awareness. The cellular boundary delimits the informational field, shielding it from some distortions or deformations caused by external environmental variables and adjacent cellular field effects. The cell membrane creates the environment in which the integrity of the information field can be protected and coherently projected. Therefore, our typical biological frame of reference of material form can be redirected towards a larger concept of information space. Phenotype becomes a manifestation of biological substrates resolving the inherent ambiguities within energy and information fields into material form. Holobionts no longer reduce to only innate cells and obligatory microbial companions but are instead considered as aggregations of overlapping Pervasive Information Fields (PIFs) wherein each constituent cell has its own basic self-referential life property. All link together enabling larger PIFs, as localized cellular ecologies and then again reiterating, in series as holobionts. Each extends in information space along its own developmental arc, experiencing and gaining vital information about the outward environment from the exchange of bioactive molecules, genetic transfers and epigenetic impacts.

## 5. An Alternative Endpoint Requires Different Mechanisms

Once biologic organisms are reconsidered as specialized forms of information fields, the linkages between the unicellular realm and eukaryotic multicellular life become more apparent. Certainly, it is understood that bacterial organisms exist in complex social and reciprocating communities [126], that are dependent upon communication and the transfer and use of information. This consequent interplay leads to complex colonial forms in which individual cells can demonstrate specialized behaviors. Ben-Jacob has determined that this effect is attributable to problem-solving via collective sensing and the use of information based upon shared environmental experiences and stored information as memory [126]. Such distributed information processing is shared throughout the information field of an estimated 10^9^–10^12^ bacteria in the colony, that transforms “sense” into a form of collective overarching intelligence. Bacteria utilize what they can to enact these changes, such as quorum-sensing, chemotactic signaling and plasmid exchange [127]. Complex colonial forms emerge through the self-organizing interplay between each individual bacterium and the colony, which can now be further pictured as systems of overlapping and reiterating individual Pervasive Information Fields inherent to each of the interacting constituents. In this manner, novel features can arise and be put to use, based upon collective problem solving that extend beyond any level of previously stored information capacity. The manner in which this occurs is best understood through the concept of stigmergy.

Stigmergy is a type of feedback loop in which any action leaves some kind of trace in a medium. Each trace, consequent to any action, incites a further action either by the individual leaving the first trace or others that follow [128]. Heylighen defined it as “an indirect, mediated mechanism of coordination between actions, in which the trace of an action left on a medium stimulates the performance of a subsequent action.” [128]. In the macro world, the best studied case is the self-organization demonstrated by the building of termite mounds.

This type of process requires some minimal level of intentionality, but only insofar as the actions are appropriate to environmental conditions. However, there need not be any explicit goals. The only base requirement is that the participants in a stigmergic system are able to send and receive information as communication.

Importantly, in such systems, there is no need for planning or anticipation, memory, intentional communication, mutual awareness, simultaneous presence, imposed sequence or division of labor, or centralized control or supervision. Stigmergy illustrates a realistic means through which information is used towards self-organization. Although stigmergy assumes that any participating agents are individually goal directed, it is independent of the goal itself. So any living entity whose goal is to maintain self-identity by sustaining a preferred homeostatic boundary condition would satisfy that requirement. Since the individual participants can have independent goals in any mixed cellular ecology, there is a natural division of labor. The variety of these participants working together build complexity, in sequence or in parallel, based on this continuous stream of information from both within any niche or shared information field, such as a bacterial colony. Since the information that is available is both direct and indirect within any PIF, under conditions in which neither sender nor receiver is necessarily clear cut, conflicts are diminished as the participants mutually edge towards consensual outcomes by always striving to remain within their own limits. This can realistically be offered as the origin of the synergy through which all tissue ecologies evolve. The hallmark of self-organization is the emergence of global order from local actions [129]. Since this organization arises spontaneously from local activities, and there is no central plan or planner, or external control, there is no organized resistance in any one specific direction and there are no actual errors being made. Only actions that constitute a general drift towards consensual outcomes in continuous reaction to epiphenomena emerge. This can result in surprising outcomes and can be considered a creative means in terms of biological expression. Furthermore, increasing collaboration becomes an effect of collective stigmergy, as an emergent phenomenon based on individual self-awareness and the reciprocity that underscores the cooperative impulses inherent within biological systems.

There is an important difference between individual self-awareness and the collective self-awareness that emerges from a stigmergic PIF. In stigmergic systems, in which information is continuously deposited as traces in the environment, the processing of information extends beyond any individual participant and extends outward into the larger environmental PIF. An example is the stigmergic organization of bacteria termite mounds with cues that extend throughout the extracellular matrix [130]. Since the information does not lie within the individual itself, yet exists within its entire sphere, stigmergic interactions exemplify the advantage of considering biological development as based on information space and Pervasive Information Fields.

One singular advantage of considering cognition as foundational to evolutionary development is that the processes by which complexity can build in the cellular realm can be compared to the manner in which humans engineer within our own sphere [19,23]. Witzany considers such natural engineering type actions as a product of communication processes within and among cells that proceed along combinatorial, context, and content specific paths through rules that have some similarity to language-like text [131]. All organisms use signs by which they can distinguish self from non-self and exchange information. RNA-based regulatory networks interact in complex ways with patterns of gene expression that can be linked to epigenetic impacts, to such a degree that it can be asked whether "evolution has learnt to learn." [132]. It is clear then that a path exists between the cognitive aspects of unicellular life that permits its reiteration in eukaryotic multicellular organisms. Ancient and fundamental links therefore extend backward to unicellular capacities so that problem-solving at the level of our own neural capacities derive from those same processes [46]. In that way, natural engineering processes can be seen as a continuum from the origin of life forward.

A number of models have been utilized to underscore the principle that individual cells and other life forms can engineer solutions to environmental stresses. Agnati *et al*. emphasize several basic principles that underscore any process of natural engineering [133]. This includes reiteration, self-consistency, and mosaic formulation by which reiterative patterns diverge to arrive at differing endpoints [133]. An additional such precept is termed the principle of Biological Attraction, an inherent drive for association based upon an “attractive” field. The effects of such a field are asserted to extend throughout biology to include interactions that are not typically considered in that manner, such as infectious interchanges [23].

Criticisms of natural engineering are generally focused on a lack of agreement as to the extent of the influence of the horizontal transmission of both genic and non-genetic materials [134]. However, heritable genetic transfers at the eukaryotic level are clearly demonstrated, including retroviral endogenizations of HIV [135], Koala retrovirus [136] or the transmission of heritable DNA from bacteria to eukaryotes [137]. Wang *et al*. have demonstrated that LTR class I endogenous retrovirus (ERV) retroelements, a distant relative of HIV, have considerably impacted the transcriptional network of human tumor suppressor protein p53, a master gene regulator crucial for primate differentiation [74]. These results demonstrate how retroelements can significantly shape the regulatory network of a transcription factor in a species-specific manner. In fact, the adaptive value of retrotransposon activation secondary to environmental stresses is frequent and contributes to the functional regulatory machinery of the cell [19] Furthermore, eukaryotic development is strongly dependent upon viral properties and impacts [131]. Such viral incorporations can be considered the long-term “domestication” of such elements and their subsequent conscription into holobionic function. Accordingly, Frank Ryan, the physician-author of Virolution [60], favors the term “symbionts” for such retroelements rather than parasites; his term implicitly acknowledges the often beneficial roles of retrotransposons.

The general reluctance to accept the primacy of natural cellular engineering might be attributable to a common assumption that honors selection as a near exclusive agency. Yet, no rejection of selection is needed. Instead, there is only a requirement to accede that there are limits to selection within a re-framed evolutionary narrative. Further, when cellular engineering is empowered as a mechanism through the agency of self-awareness as an implicit property of all living things, then evolutionary development can be viewed as the consensual enactment of cellular purposes directed towards maintaining fundamental self-referential awareness within delimiting boundaries. Retroelements then become tools, as the residual effect of infectious exchange as a form of information exchange, from which phenotypic competencies can then emerge [138]. It is known that at least half of our human genome is a legacy of past retroviral encounters, which has been termed “plague culling” by Ryan [139]. In fact, it is not merely culling as selection that matters for these infectious exchanges. They are better understood as part of a continuum of biological effects as the common currency of biological interchange on this planet. Depending on circumstances of amplitude, target, and extent, infectious interchange extends beyond the typical considerations of infectious illness to include diverse outcomes ranging from individual infection, epidemic infection, parasitism, symbiosis, mutualism or infectious latency. Occasionally, that same process yields heritable change that can then become an evolutionary event purposed towards future phenotypic alteration if the appropriate vector intersects a susceptible organism. The mechanism of all such manifestations is similar [20]. All these processes, both genic and not, become part of cellular means towards engineered solutions to environmental stresses in collaboration and competition with others. This includes the intracellular life cycle of the virome that permits the creation of new genetic paths to manipulate the environment and enact novel biological solutions according to universal self-awareness [140]. These biological combinations reverberate throughout complex genomes. By this mechanism, creative potential is fueled through the union of transposable elements and retroviral genetic sequences or LTRs with vertebrate genomes [141,142].

In such circumstances, natural selection becomes a post facto filtering agency of phenotypic differences and morphological novelties that emerge from very different impulses. Contrary to selection, hologenomic evolution considers the primary impulse of evolutionary development to be embedded information within PIFs to enable a natural and self-organizing form of cellular engineering to solve problems. Phenotype is its product. Through competitive and consensual cellular engineering processes, phenotype emerges as the reciprocating output of cellular ecologies as they reiteratively meet environmental stresses, in deep collaboration and competition with other cellular ecologies.

In any such assertions, epigenetic impacts are of salient importance. Over the last few decades, there has been a significant countering shift against the prior ingrained belief that all important genetic activity is random mutational variation within a generally static central genome [2]. This earlier viewpoint has yielded to our contemporary understanding of the larger scope of the epigenome [38,143,144,145] and its wide range of effects on genomic plasticity [146]. The functioning genetic complement of any multicellular organism is an ever-ongoing and dynamic interrelationship between any species innate cellular ecologies and an agitating epigenetic realm. A fuller extent of this epigenetic influence is now acknowledged throughout evolutionary development that fundamentally changes the epicenter of control of multicellular eukaryotic organisms beyond traditional Darwinian means [147,148]. All transgenerational genic and non-genetic heritable effects are information [149]. This is the feedstock of natural engineering processes, that then proceed by becoming a part of the Pervasive Information Field that constitutes all organisms.

## 6. Discussion

If it is considered that natural selection is not an exclusive driver of evolution, any simple assertion that evolutionary development is pluralistic suggests differences but does not represent sufficient progress. That consequential differential can be the recognition that cognition is both a point of origination as a permanent enabling mechanism and source of countervailing constraint. This standpoint is premised upon self-awareness as a state function as the conditional aspect of life on this planet. With this as its base, evolutionary development becomes the further elaboration and reiteration of self-referential cognition sustained against the stress of epiphenomena. Cognition with its own boundaries and limitations provides both release and imperative limits. Selection pertains but operates differently than typically assumed.

When cognition is the base, then the sustenance of any organism and its survival are information dependent. In the context of eukaryotic organisms, this is best conceived as a Pervasive Information Field (PIF) as the summation of the use of information to sustain self-awareness among the myriad constituents that constitute any holobiont. As opposed to our obvious material form, it is the primacy of information that matters in evolution. Information underscores self-recognition and maintains homeostasis at every scope and scale. Information space then collapses into biologic form to sustain self-referential status between necessary boundaries. Pervasive Information Fields are the systematic background through which the problem-solving matrix of eukaryotes is directed toward that goal. Derivatively then, the integrity of the information field must be deemed most consequential. It is a necessary requisite that the information field of any organism must be continuously re-centered and matched against a stream of epigenetic impacts. Without this, the information field becomes chaotic. Therefore, it can be assessed that this essential stability is achieved through the agency of the zygotic eukaryotic unicell and thereby provides the rationale for its obligatory recapitulation [150].

Within any Pervasive Information Field, the issue is not having too little information. Just as in our own lives, the disquieting reality is that most information is useless noise with respect to our own needs or purposes. In a world of pervasive information or “chatter”, in which most information is not necessarily directed towards any specific receiver, the self-organizational and cooperative impulse of stigmergic systems provides the practical mechanism for self-organizing activities within the mixed cellular ecologies that constitute all holobionts. In stigmergic systems, there need not be any directed coordination among individual players. It is sufficient that they have a means of identifying preference and act in conformity with its sustenance. Through the stigmergic feedback loop, indirect information that might be merely detritus from sender/receiver units ultimately becomes useful to one or more of the constituent players and can be utilized towards consensual outcomes without any necessity for correspondence with the intentionality of the sender.

In this manner, coordination emerges within tissue ecologies through both direct and indirect means among a wide assortment of constituencies. Most importantly, unless information is expressly directed and received, it becomes a primary form of biological ambiguity. The status of information is always uncertain and context dependent. Any sender/receiver unit must contend with that lack of clarity. Information that is directed may not necessarily be received. If received, it may never be understood. Even further, it is not necessary that any information that has been received and comprehended will necessarily yield a reaction. In that situation, ambiguity remains unless that information collapses into a response on the part of the receiver, at which point the chain of uncertainties is resolved in only one aspect and simply begins anew in another. Further too, what is noise to one entity may be actionable information to differing ones that intersect with the PIF and may not be any intended receiver. Therefore, the status of any receiver is almost always unclear with respect to the sender. As humans, musical notes as aural information are collapsed into musical appreciation in our own subjective manner. What may be noise to some is music to others. Therefore, the continuous collapse of the superimposition of possibilities is settled through a collective emergence of the resolution of those ambiguities.

Contingency is therefore contained within the variety and specializations of the individual constituents of the system, and is thereby dependent upon the flow of internal traces and external stimuli of epiphenomena. In such circumstances, stigmergy can be offered as a unifying mechanism between the quantum ambiguities in any information system and their collapse into biological expression as it reprises at every scope and scale. Physiology then becomes an enactment of self-awareness as repeatedly reinforced through multicellular stigmergic networks as a framework for maintaining both individual and collective “self” through protective homeostasis, reiteratively accomplished by the ready transfer of information.

Furthermore, it is clear that thermodynamic levels both within and outside of any cell are themselves forms of information. For example, thermal microscopy can be utilized to understand intracellular metabolism or disease incidence or the effectiveness of new cancer drugs [151]. Furthermore, the mechanical and structural properties of cells play a pivotal role in many cellular processes. While DNA may be resistant to heat, even small changes in physiological temperature compromise the mechanical integrity of the cell nucleus. Therefore, there is a reciprocal interplay between thermal cues and mechanical attributes of the cell. In this manner, physiology is properly understood as another form of information, and becomes then, part of any PIF. Physiological paths are thermodynamically efficient and lead to cellular self-organization through information transfer. At every scope and scale, self-organization becomes a directed means of protecting physiological homeostasis according to thermodynamically efficient pathways which include an entropic open system and energy dissipation [152]. Any such circumstance requires abundant information. Therefore, the ability to efficiently meet those compelling thermodynamic requirements is directly correlated to the use of that information and then manifests as the emergence of physiology from its unicellular roots [72]. As mixed cellular ecologies can be both properly defined as an informational agency and dependent upon the continuous flow of information, there is a concomitant impulse to extract an optimizing amount of energy (as the counterpart of information) within homeostatic limits from the surrounding ecosystem. In terms of cognition, this is enacted as the minimization of variable free energy [93]. Through reciprocal action, and within boundary constraints, phenotype as form and function emerge based upon cell-cell interactions [153], that is directed towards the minimization of variable free energy and the suppression of surprise (unpredictable outcomes).

When information is considered the backbone of any evolutionary frame, then information quality becomes paramount. It becomes clear then that the manner in which homeostasis is reiteratively maintained at every scope and scale requires the intercessory function of the eukaryotic unicellular zygote through which all multicellular eukaryotes recapitulate. Although not obvious, multicellularity need not have been any necessary evolutionary outcome. Intracellular engineering might have led to enormously large, efficient and capable single eukaryotic cells. Yet, that is not our known biologic outcome in the cellular realm even as it is evident in the virome. [154,155]. The question, therefore, arises as to the reason that evolution has not led to single large efficient eukaryotic cells as the dominant biologic players.

That answer lies within physiologic mechanisms based upon thermodynamic constraints that extend forward from unicellular roots. These processes are perpetually based on stable cellular principles of evolutionary development that include empowerment and constraint [84,150]. It is known that there is a crucial modulation of transgenerational epigenetic inheritance through the obligate intermediary of the zygote [156,157]. As a consequence, it can be asserted that the recapitulating zygotic unicell is the actual centrality of eukaryotic development as an enduring Pervasive Information Field that assesses current environmental epigenetic impacts within the constraints of any intrinsic genome. An essential aspect of this obligatory recapitulation occurs during meiosis as dynamic modification of transcriptional activity of sex chromosomes, histone modifications, and regulation of epigenetic programming and chromatin dynamics [158]. Epigenetic reprogramming in germ cells is critical and extends into early embryological development [159]. The variety of these mechanisms includes meiotic *trans-*sensing and meiotic silencing acting within their molecular role in protecting transgenerational genomic integrity. These mechanisms have not been fully elaborated but seem to be directed towards the prevention of the expression of rogue retroelements as novel epigenetic insertions [160]. Furthermore, those marks that are inconsistent with development and homeostasis are eliminated during morphogenesis through networks of epigenetic specificities [161]. Yet, it is also known that other epigenetic impacts continue and can affect phenotype and health [162]. Therefore, the concept of a static genome throughout the life of any organism can no longer be sustained. It is now known that there are Developmentally Regulated Genome Rearrangements (DRGRs) that alter genomes either in specific cells or during particular life cycle stages. Furthermore, these processes are widespread throughout eukaryotes [163,164]. It is, therefore, apparent that maintaining overall fidelity to a base genomic structure is necessary and this is increasingly understood as highly dynamic on both a genic and epigenetic basis [165]. As it is now accepted, dynamic genomes are the rule across the Tree of Life [166]. Nevertheless, genomic order and integrity must be still be assured, which then implicitly defaults into the unicellular eukaryote zygotic phase as the necessary intercessory agency of the self-referential unicell directed towards the resolution of quantum ambiguities against epigenetic stresses. As a derivative then, it can be asserted that selection is no longer random since there is a reciprocating interaction between constituents of the environment in a highly integrated and iterative process that prevails in evolutionary terms.

The conspicuous role of obligatory rechanneling through the eukaryotic unicellular state relates directly to the unicell as an adjudicating moment governing replication errors and the epigenome. Asano *et al*. suggest that the unicell conforms to a quantum-like master equation governing the information state of the cell [10]. In any information network, noise must be regulated to avoid chaos. Therefore, the expression or down-regulation of epigenetic marks either through meiosis, the zygotic unicell, or subsequent embryological development is the biological resolution of the quantum superimposition of possibilities stirred by ambiguous epiphenomena. Within this framework, the eukaryotic macro phase becomes something different from that which has been previously supposed. Its explicit purpose is directed towards the acquisition of epigenetic experiences that will then be placed in the context of the perpetuation and sustenance of a perpetual eukaryotic unicellular form. When considered in this manner, eukaryotic reproduction becomes the reconstitution of that eukaryotic unicellular form which has a new and potentially more flexible full range of implicates and explicates in juxtaposition to the outward environment consequent to its prior macro-organic excursion. In essence, evolution proceeds from zygote to zygote [150]. In this manner, space and time for the unicell are different from our conventional view of biological space-time. The unicell is gaining information about the external environment through a transient elaborating context within its macro form, but utilizing it according to the proscriptions of its own “self”, to which it has permanent adherency that extends over geologic time.

The eukaryotic unicell expresses only a portion of the heritable transmissions that it has received into its next macro elaboration. In so doing, it is storing information from its past. Crucially, this can become its future based upon further information that exists in biological form as the latent compaction of the superimposition of biological possibilities as opposed to those epiphenomena that may never be collapsed into biological expression. In this way, the zygotic unicell achieves a unique status as both observer and participant that privileges it to collapse the superimposition of latent states into those that are best equipped to sustain its homeostatic “self” over very long-term environmental cycles. Any re-elaborated macro form is therefore equipped to deal in flexible terms with widely shifting though temporary environments. By this means, the unicell escapes any rigid or traditional view of biologic space-time through the entanglement of pervasive information systems that are both its own but also part of the outward environment with which it has contact through both direct communication and non-local correlation. This intersection of both inner and outer information spaces is reiterated in the macro form and explains a wide range of biological actions. For example, the avian magnetic sense organ used to detect magnetic fields operates on a quantum basis via entanglement with molecules acting simultaneously at a distance. The final state of one molecular action of that type is determined after the fact by a subsequent one without apparent connection [96]. Monarch butterflies and fruit flies use similar quantum effects in navigation, and plants are dependent on quantum processes for photosynthesis. Instantaneous muscle coordination over a scale of distances over nine orders of magnitude by the coordinated splitting and release of 10^20^ molecules of ATP in all animals is another example of non-local correlation in biologic form [12].

This entanglement principle is further exemplified through overlapping information fields in the context of complex interrelationships between the unicellular zygotic phase, the post-zygotic embryonic development, and the macro form. Recent research has elucidated both the importance of the unicellular phase in the frog embryo (*Xenopus tropicalis*) and overarching maternal control of frog embryonic transcription [167]. That overlapping control network is depicted through epigenome reference maps within the unicellular zygote that are partially formed by the maternally defined epigenetic regulatory space, instilled within islands of hypomethylation enacted by deliverable proteins and maternal RNA that are part of maternal cytoplasm. This maternal overlap predominantly controls gene regulation in the frog embryo through the first twelve divisions but its influence also extends into the later regulatory space. A similar process is present in mammalian embryos, though the exact timing patterns differ. In all instances, however, information undergirds development. Therefore, information space is a property of the state function of self-awareness implicit to all living things and exists as a crucial entanglement between variational free energy as self-organization derivative of thermodynamics that eventually resolves into biological form.

In like manner, the concepts of information space are essential to understanding embryonic development. Kirschner and Gerhart detail embryonic spatial mapping and developmental compartmentalization as keys to regulatory control [168]. Since there are no simple anatomic boundaries, biological expression results from highly coordinated communication among linked developmental compartments in a system that is best understood as consisting of overlapping transient developmental environments. Further yet, such communication could only exist within the context of an overarching information field that controls the timing of that development. The obligatory recapitulation of the eukaryotic unicell is therefore a crucial centering of that essential PIF to regulate the expression or down regulation of epiphenomena accumulated during the prior macro elaboration. Absent such a mechanism over successive generations, there would be heritable chaos. There would be a similar potential for damage within any intrinsic genome if there was no superbly efficient mechanism for faithful DNA replication and the policing of replication errors. It can, therefore, be asserted that there is an information field, continually centered and adjusted, that permits the elaboration of the linked compartments that are both contiguous and distant that enables embryological development.

Under these circumstances, the connection of the macro form to its re-elaboration is changed. The macro form, as its own PIF with its exclusive environmental experiences and acquisitions, is a contributory essential to the unicellular zygote. However, its next macro-organic elaboration is altered through the adjudication of the self-referential zygotic phase whose residual influences redound throughout the embryological arc and beyond. As a corollary then, the concept that evolution relates to simple gene frequencies can no longer be sustained. The obstacles to this assumption are now many, not the least of which is that our entire concept of the composition of a gene as an anatomically discrete zone of DNA has been completely reappraised since the formulation of the Modern Synthesis [169]. Furthermore, epigenetics is entirely centered upon a discordance between simple gene frequency and further genetic expression in biologically active systems. Nor does any simple notion of gene frequency realistically incorporate holobionts into its purview as evolutionary entities in which microbial genetic material is at least 100 times greater than the innate genetic information of any macro form [170]. Nor does it incorporate any theory of complex limiting behavior of multi-locus genetic systems as that relates to the interactions of ecosystems or constituencies within ecosystems [45]. Therefore, it can be maintained that the differing contributions of genic and non-genetic inheritance impose a necessary fresh conceptual framework that is not merely related to raw genetic frequencies, or even to genes alone. The alternative requirement requires an understanding of the flow of energy as information in biologically active materials harnessed and constrained into a consistent and inclusive framework [171]. As such then, genes can be understood as not merely “units of inheritance” but as an emergent expression of information space reciprocally dependent upon cellular processes and dependent upon information inherent in bioactive molecules and extrinsic epiphenomena [172]. In such circumstances, everything depends on everything else, and phenotypes then become emergent properties of a larger overarching biologic information system that is inclusive of heritable proteins, lipids, and cytoplasm and largely extends beyond nuclear DNA [173]. Therefore, when energy and information are considered as differing aspects of an entangled equivalency, then any PIF is thereby inter-related with any bioactive energetic field with which it has contact, in a construct that might be considered an enhanced Markov blanket as interconnected nodes of diverse parentage, connected to the network but still retaining conditional independence. In this manner, the nodes in any specific PIF retains some aspects of conditional individual intentionality in reaction to stress, while remaining within other overarching fields. In hologenomic organisms, energetic processes such as heat dissipation minimize variable free energy and propel self-organization which can then be understood within a context of entanglement between information, energy and biologic substrates. An explicit example is the propagation of neural activity by endogenous electric fields [174].

It can then be maintained that within pervasive information systems based upon self-referential cognition, genes serve to maintain the information system and then, in turn, are also reciprocally being served. Any explanation of biological evolution in terms of gene frequency refers to outcomes rather coherent process when cooperative mechanisms, collaborations, and reciprocity have sway [175]. Therefore, it can be asserted that it is not merely genomic integrity that is re-centered through the recapitulating unicell, but more accurately, an overarching Pervasive Information Field that enables every organism. It is during this phase that those permissive and involuntary modifications acquired during the re-elaborating macro phase are readjusted towards the longer-term moving average of the dominant environment trend. Absent such a process, any genome, all cell processes and the information fields that control them, would become increasingly chaotic. The easily overlooked implication of our contemporary understanding of the large extent of the epigenetic influences experienced in every phase of our life trajectory is that a re-centering mechanism towards a longer term environmental average is essential. Absent this, organisms fatally skew towards temporary aberrations.

A crucial question then applies. Can the concept of a Pervasive Information Field substitute within biology for actual physical form? Certainly, such an overarching field exists since its activity is clearly apparent in the developmental stages emanating from the delimited form of the unicellular zygote. As an example, the embryological spatial map has no anatomic correlation with subsequent form [168]. It is clear then that information content has primacy over form throughout the recapitulating reproductive cycle and its immediate postzygotic development. Therefore, it requires only a little imagination to consider a dominant PIF that is its own specific form of sender/receiver space-time whose existence is implicit as an overarching archetypical entity entraining energy. In such an instance then, any physical organism becomes a manifestation of biologic expression as a transient flux agent of a particularized information field. In essence, all multicellular eukaryotic organisms become transient informational subsets of a larger dominant eukaryotic PIF. Each extends outward into the environment and intersects with other information sets as a reciprocating constituent of the larger environment.

Within this model, fitness is a transient enforcement of one of the superimposed possibilities of any PIF, as part of a subset of the full spectrum of a dominant eukaryotic PIF. This bandwidth subset then gets briefly expressed as phenotypic form. Natural selection is a tautology since its action is a post facto concentration on phenotype that is a derivative expression of a larger encompassing overlapping Pervasive Information Field. Any macro form is merely a temporary fraction. Therefore, at any moment in time, current biologic form is the settling of the superimposition of possibilities from a larger dominant unicellular eukaryotic information set as a temporary manifestation of a narrow range of specific informational subsets. Therefore, whatever set is not currently expressed or has been eliminated is by definition, “fit”.

Therefore, it can be asserted that there is a dominant Eukaryotic Pervasive Information Field inherent to that fundamental cellular form as opposed to that of Bacteria or Archaea, and further, that is not a direct object of selection. It exists above our ordinary understanding of selection. However, any PIF subset expressed as a eukaryotic macroorganism is acted upon by selection. Therefore, selection is an agency of temporary bandwidth flux of a larger information set that is perpetual as a Eukaryotic life form. Selection becomes the temporary settling of a range of implicates within the PIF of that master Eukaryotic cellular domain as an information subset of latent potentials resolved into biological explicates.

Therefore, reproduction is more than a means towards reiterative phenotypic expression. Sexual reproduction is the best means of re-centering any PIF through meiotic averaging. Eukaryotic multicellular organisms are a representation of a bandwidth of an overarching PIF as a derivative thermodynamic entity and information subset. Therefore, any organism as a material entity is the physical embodiment of a unique PIF subset that must stay centered within a long-term environmental trend even as it flexibly deals with shorter term and transient environmental circumstances. It can be suggested that any transient macro elaboration is a necessary and limited environmental taste akin to the difference between daily weather and long-term climate trends. That longer term consistency channels through the self-referential agency of the zygote unicell and its own PIF subset.

In any system of nested ecologies that are constitute holobionic organisms, it is plain that order must be maintained. In biologically active terms, this is an immunological expression [23]. Undeniably then, the only means by which holobionts can exist as an explicit reality is through an active immunological compact. Further, the core purpose of immunology is self-recognition against “other”. Therefore, cognition as a condition of life is dependent from its inception upon immunological means to maintain “self” within an active biological frame that must continue in a reiterative manner throughout evolutionary development. Indeed, this is obvious. Without effective immunologic mechanisms, there would be only a single biologic organism undifferentiated from any others. Yet, the impact of immunology on the entirety of any evolutionary narrative and the centrality of holobionts as multigenomic consortia governed by immunological imperatives has only just been recognized [3,23].

## 7. What Does This Mean for the Modern Synthesis

In 2010, Lewontin noted that the standard formulation of evolution by natural selection does not explain the actual forms of life that have evolved and further contended that there is an immense amount of biology that is missing from Neodarwinism [176]. Any proposed justifications must, therefore, grapple with the central dogmas of the Modern Synthesis and provoke essential questions. Is evolution primarily a narrative of natural selection? Does it proceed according to strict gene frequencies? Is it a merely random process? Does Crick’s Central Dogma asserting a unilateral direction of the flow of biological information from DNA to RNA apply? Any worthy answer must concede that any facile assertion of exact opposites is also inappropriate. As with all complex and well-calculated concepts, the inherent depth of their profundity is that simple contradictions are not themselves necessary absolutes. Therefore, any oppositions are not unyielding negations but are instead directed towards a fuller understanding of evolutionary development within a complex schema.

For some, the search for satisfactory answers has been to recast dogma into a more flexible form. Müller suggests that evo-devo is “a causal mechanistic approach towards the understanding of phenotypic change in evolution” [177] and is no longer just about gene frequencies. Yet, that frame is still deeply selection dependent, even as it denies that genes work in a linear fashion and are subject to extensive feedback from many associated players within developmental constructs. Mattick emphasizes the importance of intergenerational epigenetic inheritance and a prominent role for RNA regulation of the epigenetic state [132]. He conclusively dismisses Biology’s Second Law, known as the Weismann barrier. Somatic cells and germ cells are not exclusive from one another. Further then, since RNA editing can alter genetic code in a context dependent manner as an epiphenomenon, then phenotype becomes that dynamic product. Therefore, any long-held belief about the absolute centrality of DNA must be set aside.

The concept of “facilitated variation” has been proposed as one solution to the problem of developmental pleiotropy [168]. In that perspective, core processes remain intact but the regulatory components determine the extent of variation, which is still based upon random mutations and subject to standard selection mechanisms. Since only the regulatory side experiences that variation, theoretically then, only a few mutations in that space would be needed to generate novelty. In effect, the number of unlikely steps is theoretically reduced. Somehow, organisms are considered “poised response systems” ready to make changes that they are “prepared to make”. Still, however, within facilitated variation, those changes are not still directed or enumerated beyond selection and random variation.

The Predictive Adaptive Response hypothesis has been offered as a differing alternative [178]. This is a form of developmental plasticity in which early life environmental experiences can influence fitness later in life, and could theoretically induce fixation of epigenetic markers. This is a more pluralistic form of Neodarwinism, still centered on selection and limited in scope as an explanation of developmental novelty. Yet, its base assumption importantly construes that organisms have the capacity to anticipate future fluctuating environmental conditions and act upon that forecast. In Predictive Adaptive Response, the delay between the incursion of epigenetic impacts and the induction of phenotype is a form of "forecast about the future conditions of the external world." [178]. Hologenomic evolution also asserts a predictive capacity but offers a differing interpretation. In cellular terms, predictive power is offered through the agency of the recapitulated zygotic unicellular form that permits the adjudication of epigenetic markers to meet long-term environmental stresses as opposed to transient ones. In this circumstance, the “forecast” is a prediction of the future environment only insofar as it recognizes that there is a dominant longer term environmental trend that will ultimately reassert itself compared to transient environmental extremes. The predictive capacity of the unicell can, therefore, be understood as its ability to match the shorter term environmental exigencies into the context of the more consequential and enduring longer term trends. In that sense, the zygotic unicell contains information from both the past and future. The latter remains in latent form within the PIF of the unicell and is then used to discern the activation of epigenetic marks or the down-regulation of others.

In a review of the integration of evolutionary biology and physiology, Noble *et al.* provides an overview of the burgeoning extensions to the Modern Synthesis [179]. In particular, the role of epigenetic horizontal transmission is now viewed as displacing the absolute primacy of vertical descent. Further, the traditional congruence between genotype and phenotype implicit within the Modern Synthesis is no longer regarded as tenable against a broader understanding of heredity in which concepts such symbiogenesis and natural genetic engineering are now offered as consequential adjustments.

Each of these constructs nudges evolution in more contemporary directions in which a gene-centered and selection dependent formalization of the Modern Synthesis must yield. What might be considered instead? Shapiro clearly outlines one essential difference [180]. Certainly, the flow of genetic information is not exclusively from DNA outward, thereby vitiating Crick’s Central Dogma. Additionally, the concept of the gene as a discretely localized region of DNA code is inaccurate. Further yet, any specific “lock and key” mechanism between molecules and biological interactions must be reappraised in light of the flexibility of molecular subdomains [19]. It is also becoming apparent that genetic mutations do not account for genomic change compared to other processes such as natural genomic engineering. But even this is not a sufficient. The cell should not be viewed in purely mechanistic terms [181]. Instead, the entire cell must be regarded as an informational system in which decision-making is its central function. An important aspect of this reconsideration is that intracellular decision-making processes or decisions among cells are resolutions of biological ambiguities sustained from environmental stresses in furtherance of critical homeostatic balance. Information transfer is the backbone of cellular processes and takes forms that have not been typically considered as such. For example, horizontal gene transfer is commonplace and not restricted solely to prokaryotes as assumed under the prior dogma of separation. Examples of transfers from prokaryotes to eukaryotes, such as the horizontal transfer of terminal proteins between prokaryotes and the eukaryotic nucleus are documented [182], as well as the horizontal transfer of genetic material across species boundaries as a form of niche construction [183]. Therefore, as a necessary correlate, the underlying rules governing information transfer between cells is dependent upon immune status represented through major signaling molecules as part of the information system that governs all aspects of cellular well-being [184,185].

Once it is understood that information fields rather than phenotype underlie evolutionary development, it follows that the biological rules in the unicellular and viral realms do necessarily apply to the eukaryotic one. The fundamental information spaces are perpetual. It is therefore not surprising that developmental strictures are based upon the disciplining agency of obligatory recapitulation through the eukaryotic unicellular form and the consistent imposition of overarching immunological rules that also recenter within that unicellular phase. From this, it follows that despite macro appearances, our planet has remained firmly anchored in cellular life across eons and remains so even now [71,72,150]. Examples include ribosomal translation [110] or the glucocorticoid receptor protein whose ancestral form permits modern conformational flexibility [186]. Therefore, the past perpetually recapitulates through the unicellular form, whose forecast of the future is its knowledge of the past in geological space-time. Within this greater narrative, genes serve, and then as constituents with their own biologic “selves”, are in turn being served. In consequence, it becomes apparent that there is a suppleness to evolution that eludes any mere conformity to a narrative based upon selection as an effective exclusive agency.

In an evolutionary system predicated on information exchange derived from energy transfers, selection is a byproduct of that information system, not its driver. Placing information at the center of biology is not unique [88], nor is considering communication as universal to life and or as a directed means towards problem solving [83]. However, placing it within the context of an overarching awareness of preferential status as a derivative of homeostatic imperatives represents a significant differing perspective [23]. Differing too is an appreciation that biologic form is preceded by information space, derivative of energy transfers, as its own matrix both intrinsic to and still distinguishable from any material biologic entity.

Also separate from prior theory is the assertion that information space and any resultant self-awareness are intrinsic properties of a system in which ambiguities are consistently resolved by consensual and collaborative holobionic players as the proper end point of eukaryotic development. Implicit in this differing viewpoint is the acknowledgment of macro-organic structures as a unique adherency of confederated life united through information space. This extends beyond presuming that microbial life is merely affixed to a host scaffold but is instead predicated upon a framework of such organisms as intimate and profound seamless interconnections of cellular and non-cellular constituencies. Therefore, any microbial eukaryotic cohort is not simply appended as a scaffold. Instead, all participants are part of a complex, transient, and dynamic life form arc. It is our instinct to appraise the differences between microbe and innate cells of any eukaryote and dismiss the requisite dependencies. In a reiterating manner, eukaryotic evolutionary development becomes a comprehensive whole. Its common currency is the flow of information as communication towards the preservation of self, that is reciprocally then in service to all cohabiting living entities in eukaryotic cellular confederacies. Our biological interactions on this planet are all directed towards those mutual non-exclusive aims.

The dynamic patterns through which these biologic principles are entwined are well known though typically casually misunderstood as merely infectious interchanges or dispassionately denoted as “horizontal genetic transfers”. Instead, the broad range of infectious interactions, encompassing individual infection, epidemics, parasitism, mutualism, symbiosis, latency and evolutionary genetic interchange are means by which self aware biological entities communicate, collaborate and compete. All biologic manifestations then become derivative of a singular overarching principle of information transfer directed towards the maintenance of self-referential preference [23]. Within this necessary linkage, it is also clear that the rules are always immunological. Such a declaration is actually self-evident. Successful reproduction depends upon self-similar recognition through immunological compatibility as opposed to dissimilitude. Reproduction, upon which natural selection depends, is absolutely under girded by immunological phenomena [23]. Clearly, in any modern context, immunological factors determine reproductive success more than access to mates or any other macroscopic metric. Therefore, immunological distinctions, rather than traditional measures of fitness, define the operating characteristics of our biologic system. Of course, immunology is also simply a variant expression of a larger organismal information system. Natural selection certainly pertains, but only as a reproductive post facto filter. Therefore, selection is a derivative function of the immunological enforcement of self-awareness as the essential property of an information system in which immunological action is itself simply another form of information and communication. Further too, it is indisputable that immunological recognition is itself a differing aspect of cognition that guides cellular decision-making within any information matrix [187].

There is another differing feature of any information matrix that impacts evolutionary development as a problem-solving mechanism. All creative cellular inter-reactions are purposed towards the resolution of biological ambiguities. However, the context in which this can occur is one of biological relativity in which neither causation nor observer status is fixed. Within such a system, control is iterative and disseminated, enacted layer upon layer. Consequently, decisions are enlivened across linked networking constituencies to reach consensual solutions to environmental stresses. This is the process by which separable living entities become holobionts. Therefore, in hologenomic evolution, causation and observer status simultaneously exist at multiple levels in a manner that confounds any simplistic Darwinian narrative. The proper frame is then clarified, a perpetual sphere of Bohmian implicate and explicates [188] pre-testables both expunged and renewed, always in transit towards its further self, ever arriving, never leaving, overlapping transient losses and gains, constructions and deconvolutions, but always a perpetual cellular/viral realm of self aware entities in service to self and eukaryotic wholes. Within this construct, it would be mistaken to assume that all information is useful as opposed to noise. And further, it would be equally incorrect to assume that all information is welcome. Indeed, many infectious interchanges are explicitly the latter unwelcome information that returns to the eukaryotic unicell among survivors and then becomes a critical aspect of the recapitulating information field.

What then is the creative aspect of evolution within that frame? Biology uses its own tools in the selfsame manner that we, as humans demonstrate within our own frame: collaboration, co-dependency, and competition are directed towards solving ongoing stresses in a continuous stream of enacted preference. This is our own human narrative, just as we construct cities, resolved at the cellular level [19,23]. Necessarily then, our human use of both inorganic and organic materials is our particular biological manifestation of cellular impulses brought forward from eukaryotic unicellular origins and then expressed within our boundaries. Plainly, we, as humans, are cellular, creative and cognitive entities, derived from and faithful to our evolutionary roots.

Might all this be random? When that answer is properly framed, it becomes quite clear. Any system in which creativity is a means towards environmental problem solving is primarily non-stochastic. However, it is not that random inputs are of no consequence. Crucially, though, in the context of the intimate and shared connections of any holobiont, random inputs are channeled towards problem-solving. Therefore, random epiphenomena can be utilized in some cases or resisted in other circumstances at many levels and then, most particularly, at the level of the adaptive immune system. Yet, other epigenetic incursions cannot be resisted and demand a place. They must, therefore, be accommodated and then may become yet another addition to a capacious eukaryotic genome and adjust its particular PIF. By this process, and at each moment, the range of implicates consequent to any variety of epigenetic incursions as experienced by any multicellular entity is directed beyond random towards resolving present and future cellular biological ambiguities in the face of environmental stresses. When that process settles into any explicate form, natural selection then has its sway.

Any dispute about the relative importance between Lamarckian forms of horizontal acquisition of heritable information as opposed to vertical descent considered primary within Darwinism is also then re-framed in this new construct. Each serves and differs, but both are purposed towards cellular needs and imperatives. Most particularly, though, the central action of evolution is no longer invested in the macro form but instead remains constituted within the cellular one. The eukaryotic life form remains anchored within its cellular origination as an iterative form in which it transiently seeks information from the outward environment and then returns it to the unicell. In that manner, terminal addition becomes non-stochastic and a form of cellular creativity. Phenotype emerges through this narrative.

Newman and Müller have defended that major evolutionary developments such as the origin of the vertebrate limb emerge through a “bauplan” based on an interplay of genetic and epigenetic processes that should be considered as self-organizing properties [189]. If this perception is endorsed, then a further aspect can be advanced. That “bauplan” is the Pervasive Information Field that defines any form of life. In the eukaryotic life form, that PIF “bauplan” is perpetually adjudicated through the obligatory unicellular zygote as it spills through the embryological compartment map and undergoes sequential developmental reiteration. As Newman and Müller note, selection has its part but does so secondary to other originating processes by adjusting and stabilizing forms. Selection then, according to Newman, is not needed so much as thermodynamics and self-organization. In the hologenome, cognitive constituents make decisions between implicates and explicates, according to their homeostatic needs as a further reflection of their self-referential state. In so doing, new homeostatic boundary conditions are set, at their limits, that become the thresholds of creativity. Evolution then flows from bounded sets of implicates based upon internal cellular dynamics and epiphenomena into explicates as biologic expression. Evolutionary development elaborates and reiterates from that in the continuous process of sustaining cognitive self-awareness against the stresses of epiphenomena of all types. As De Loof [84] has stated, it is problem-solving activity that precedes selection. However, crucial to any such problem solving is the information field that permits communication that can be directed towards resolutions. This is the means by which interactions are enforced between agents that have traditionally thought to be uncoupled [125].

With the foregoing as a central perspective, a fresh synthesis can be discerned that is distinguished from the biological materialism of natural selection theory and must be directed towards quantum concepts. Such a thorough reconsideration can be regarded as a cognitive entanglement theory. If there is to be any acceptance of this contention, there is only a single requisite. There must be an acknowledgment of an inherent entanglement between physics and biological entities through the thermodynamic state function of self-awareness. In a manner yet to be determined, energy acquires the faculty of information by which it senses both its direction and its preferred state within a given set of boundaries. In its most basic terms, this is a vectorial function, that is not dissimilar to Feynman’s Path Integral Formulism of Quantum Field Theory as indicated in his conceptualization of time as a vectorial sum of histories [190,191]. In this construct, any particle (or entity) can travel between points along an infinite number of paths, all of which has a certain probability that can be described as a wave function. As these wave functions spread through space, they can cohere or interfere with each other, and the sum of all the resultant amplitudes results in the final discrete path that it eventually follows [192]. If a similar line of inferential reasoning is used in biological terms, biologic space-time represents overlapping information fields. It thereby proceeds through quantum entanglement with other energetic vectors, each a sum of its histories as implicates and explicates. It is through this entanglement that self is derived and then, ever and always, continues to define the biological interactions between the self-referential entities that are then, by definition, alive.

It has certainly been skillfully maintained by others that biological processes can only be understood within a quantum frame. Ho has emphasized that thermodynamics in biologic terms fundamentally differs from the linear thermodynamics of Boltzmann [193]. In those terms, it is not dependent upon the acknowledged genetic or biochemical processes, but rather upon quantum coherent fields through which biological action coordinates. Life has been pictured in that frame as a far-from-equilibrium coherent photon field in a range of frequencies. The differing components of the organism, each with their unique characteristics, nevertheless synchronize together through quantum coherent fields. McFadden too emphasizes quantum effects through decoherence and the ability of cells to measure their quantum status [192]. In information space, these quantum thermodynamic field considerations unite into a faculty of quantum assessment of energy through a phase transition whereby energy becomes information by knowing its direction and status instantiating self-awareness as a condition of life. There are, however, substantial inherent differences between the means by which information systems in living things can be compared to theoretical models. Shannon information systems presuppose random variables as they pertain to the source of information independent of the object; Kolmogorov Complexity (algorithmic information theory) maps objects through sequence length seeking to determine the shortest sequence that transmits the information and then comes to a halt [194]. The limitations of theory can be readily appreciated in biological circumstances since information may not be random nor is there any necessity for information to follow either the shortest path or proceed by the most efficient means. Yet, a framework of Shannon information is still important: information is understood as inversely related to ambiguities and the extent to which they are resolved. This fits extremely well into any concept of an informational field as a probabilistic subset in which some aspects settle into biologic form and others do not. Further too, both theoretical models attend to mutual information processing providing for shared information; one object offers information about another, whether random variables in Shannon theory or sequence information in algorithmic theory. However, there is an important implication of both models with respect to that sharing and transfer; reciprocation is its implicit derivative. As Grunwald and Vitányi assert about information systems, "In an appropriate setting, the former notion [one object offering information about another] can be shown to be the expectation of the latter notion." [194].

That such quantum processes underscore human cognition has been advanced as essential [195]. The advantage of this frame is that these processes are being actively researched both within neuroscience and physics [75,196,197]. Therefore, a full range of experimentation and research can be devised, yielding the predictability to evolution that others, such as Morris have sought [198]. It is only in this manner that any open-ended and indeterminate process such as Neodarwinism is subject to testing and refutation.

When entanglement as information sharing is considered as the base circumstance, niche construction can be better understood as its reiteration at every scope and scale in which the traditional concepts of proximate *versus* ultimate causation might be forsworn [199]. Although niche construction is traditionally considered as the expression of genetic and acquired semantic information, it is also seen as a process through which organisms discriminate and adjudicate environmental stresses [200]. It is a clear imperative of niche constructions that organisms must modify environmental states in a systematic and directional way. The critical point is that niche construction endorses organism–environment complementarity and not simply the Darwinian selection of genes. Niche construction is specifically a concept of the entanglement of living entities with each other in reciprocation with environmental impacts. It is through this responsive interaction that directionality derives [1]. In this manner, niche construction theory in its varied forms is the bioactive representation of cognitive entanglement theory.

It is certainly understood that our perceptions of the external environment or the internal environment are not absolute. Our structure is that of entangled constituencies, with complex internal and external surfaces as part of our organic makeup. Indeed, within any frame of entanglement within the complexity of holobionts, the concept of causation itself becomes entirely artificial and any divide between proximate and ultimate causation must yield. As Noble asserts, there is no privileged level of causation and the concepts of proximate-ultimate are best understood as metaphors [201]. The macro form is a linked confederacy. Cause and effect are disseminated among cognitive players both in direct and intimate contact but also through non-local correlation though separated by distance. Yet all are still in contact through a system-wide flow of information. In these terms, genomes do not exclusively determine any organism but participate as entangled ensemble players among others in which the Pervasive Information System is the overarching conductor. It is not genes alone, nor any “milieu intérieur”, or the environment as exclusive agents, but an entangled interplay, based upon individualized self at all levels executed through immunological rules within a world whose only consistency is ambiguity.

Therefore, hologenomic evolution is not merely another terminal addition to Darwinism. Nor is it an antipode. It is both differing and complementary, describing the limitations of natural selection, but acknowledging that selection influences reproduction and population frequencies. It accepts that variation underscores our evolutionary narrative but insists that its mechanisms and means are beyond random circumstance. It originates from its own platform of self-awareness as a condition of life, but also embraces replication as a reiteration of self while noting an entailing necessity; self-awareness as an intrinsic property must precede it. There is room then within contemporary evolutionary biology for creativity and determinism. Not towards any explicit outward endpoint, only toward the continual perpetuation of primal unicellular forms. The discomfiting issue is plain. To what can we ascribe the perpetuating success of the eukaryotic form? Random or not? A creative response to environmental exigencies or not? On a theoretical basis, this is the entire crux. If we absolutely knew the answer to those two questions, then the rest is detail. Cognitive-based hologenomic evolution suggests its answer. There are non-stochastic forces that can be identified in evolutionary development. Therefore, even though random actions remain crucial, the system is then, by definition, no longer random. That reason can be directly ascribed. Eukaryotic evolution is determined by cognitive eukaryotic cells responding according to their scope and scale to environmental stresses. Their reiterative cooperative and reciprocating reaction at separable, yet interlinked scales is our macro-evolutionary narrative.

## 8. Conclusions

Since cognition is everywhere apparent among biologic organisms, then any biological system must be built upon it. Any organism as a thermodynamic dissipative entity becomes an information transfer mechanism that resolves into physical expression by minimizing its variable free energy based on the settling of ambiguities according to quantum proscriptions [96]. The center of all such activity is information transfer, enacted through biological organisms as communication among self-aware participants. Evolution can then be properly defined as an information transfer system and can no longer be represented as primarily related to either material biological form as phenotype or natural selection acting upon it.

The line of reasoning that extends to this conclusion is quite direct. Energy comes first. Information is its derivative as a specialized form of energy in context. Physical form then follows. Necessarily then, physical space is subordinate to information space. DNA, RNA and all the various transcription factors and bioactive molecules are intermediaries of information storage and transfer, just as macro-organisms are acknowledged forms of energy storage and transfer. Since the epicenter of communication as information transfer is ever and always enacted at a cellular level, cellular imperatives become the primary driver exerted towards the maintenance of self in homeostatic concert with the environment. Any information set that produces self-awareness is a unique Pervasive Information Field. From that moment of delimiting instantiation as a circumscribed set, any PIF then becomes the sum of the histories of that field and also the summation of its latent potentials to meet environmental stresses.

It has been recently demonstrated that the history of a photon is not one of fixed chronologies but is instead its simultaneous multiple chronologies that are all intertwined as if all had been experienced [202]. In biological terms, the zygotic unicell is the sum of its chronologies that always represents more than its current physical form. It is ever the summation of latent markers that might have permitted the probabilistic settling of alternative actualities. All can exist coincidentally within the zygotic unicell in near equal terms, some expressed and others not. Some of these implicates are in fact prior histories that had yielded prior phenotypic manifestations. They were transient biological actualities as specific phenotypic forms but are no longer so. In the unicell, their equality is that they are each simply differing quantum paths and alternative resolutions within a field set that represents the summation of all those possibilities and thereby simultaneously includes its past and its future. Crucially, physical form as any might apprehend it with our own senses is subordinate to that information space as the sum of both the light and shadow of every living thing.

In the circumstances of the hologenome, the entanglement is more complicated. Each of the constituents that form a holobiont has some degree of independence. Each has its own Pervasive Information Field and is, therefore, its own unique sum of histories replete with its own individual latencies and actualities. This is precisely the type of entanglement that can yield biological creativity. Potentials and actuals entwine in problem-solving through creative solutions to meet exogenous and endogenous environmental stresses. The sum of all histories is within each and can be rendered from thermodynamic principles into active biological expression or latency, both of which are well represented in biological systems. Latent markers remain as unexpressed potentials that might blossom only when specific triggers and criticalities eventuate. In iterations then, holobionts are enacted as linked cellular ecologies whose constituents are themselves self-aware participants with their own intrinsic PIFs. The maintenance of the perpetual and superimposed eukaryotic PIFs supports these macro entities through the assurance of the continual re-centering of the basal integrity of a dominant Eukaryotic PIF through an obligatory unicellular form. Indirectly then, genomic integrity is maintained *versus* the outward environment. Importantly too, the holobionic nature of all multicellular eukaryotes and its vast interlocking relationships with the microbial sphere are governed by immunological interactions upon which self-recognition and the integrity of biological information depend.

Therefore, eukaryotic evolutionary development is properly considered a self-referential creative process in opposition to the persistent onslaught of epiphenomena. It is expressed in terms of communication, collaboration, and cooperation, just as well as competition. In hologenomic entanglement, it is not natural selection at the macro whole as our senses contend that is the controlling agency of evolution but a differing impulse: preservation of self-referential information fields at every scope and scale, as mediated by states of homeostatic preference. Reproductive frequency still pertains but is only one aspect.

Morris emphasizes that evolution tends to converge towards similar forms and structures, despite differing points of origin and even using differing biological substrates due to adaptive constraints [203]. Any such channeling is best understood when those limitations are imposed upon information fields and their subsets that exist within their own countervailing restraints. This enables the unification of quantum concepts with emergence and convergence into a single comprehensive whole. Order in biological terms is spontaneous, but only in the sense that it derives from an instantiation of a property of self-awareness that is a part of the thermodynamic scale according to a harmonic that is not comprehensible in our current terms. The origin of self- organization may not yet be absolutely clear, but its existence is not in doubt throughout our biological system insofar as both divergence and convergence are simply differing aspects of the flow of information of any adaptive landscape [204].

What then is the differential crux between standard evolutionary theory and any hologenomic transformative one? Perhaps this is best appreciated through the illustrative manner in which Adam Smith, in *The Theory of Moral Sentiments*, (1790) discusses the operating presumptions of any human governmental or legislative “man of system”. He states “(such a person) does not consider that in the great chess-board of human society, every single piece has a principle of motion of its own, altogether different from that which the legislature might choose to impress upon it.” [205].

Contemporary research justifies an assertion that human society demonstrates many echoed reflections of its entire evolutionary journey. Consonant with that principle, as enacted at every scale even to the present moment, we remain in a continuous struggle against any invariable imposition of any “man of system”. If that dynamic is deemed accurate, then there is no permanent overarching Darwinian “man of system” operating in any macro plane. Otherwise then, we too, would be its perseverating reflection and accept its imperative control to rule our lives. Instead, it is our individual human impulses that govern our creative capacities that permit our collaborative endeavors. In like kind then, individual self-aware cellular and non-cellular constituents unite towards confederacies of creative expression through the perpetual agency of the eukaryotic cellular macro form, either tentatively or intimately, and collaborate in an outward elaboration to taste the environment. In so doing, all the co-aligned participants are thereby changed through that transitory embodiment. They return through obligatory reiteration to the eukaryotic unicell as a mediator of a larger hologenomic emergent “self” in both willing and obligatory co-alignments that form all macroorganisms. This perpetuation is assured through the eukaryotic unicell as a reiterating continuous loop from macro form to unicell and back once again, thus preserving the same self-referential exactitude from which it emerged. This is eukaryotic life properly appraised. Associated constituencies of individuals, each with their own “principle of motion”, participate in mutual concert and apposition in a transient arc of conjoined life. Phenotype thereby emerges as consensual form. It is the creative biologic expression of the aggregated homeostatic requirements of the individual constituents as they serve themselves and the linked constituencies of confederated ecologies that together represent a holobiont. Each constituent has its own “principle of motion” in service to itself and, in turn, in service to the whole. That hologenomic reality, as the product of co-linked bioactive individual entities, provides a consistent impulse that can be united into conjoining force yielding biological expression that is always schooled by the reactive imperatives of endless epiphenomena. At every scope and scale, this quantum summation is reiterated through contrasting shades of collaboration, codependency and competition and reciprocation. Constraints are present too: immunological boundaries reinforce self-recognition and are further resolved through the consistent disciplining filter of selection.

So then, what is hologenomic evolution if not a further appendage of Darwinism and competitive natural selection theory? The primary differences are clear. Hologenomic evolution, in which cognitive entanglement has primacy, is the settling of ambiguities that arise from self-awareness. Living entities utilize information and communication to temporarily resolve ambiguities to sustain self-awareness that arises as a state function derived from thermodynamic principles. This is both the condition of all living things and the property through which it can be defined. All further evolutionary steps are then subsidiary. Importantly, though, self-awareness perpetually dwells in uncertainty. In contradistinction, the Darwinian frame assumes “knowing” concrete form and discrete place in apposition to others. Cognitive entanglement theory of which hologenomic evolution is subordinate embraces the altered frame. In all biological circumstances, uncertainty is the ruling biological constant. Therefore, any system of evolutionary development must specify a process that enables the resolution of quantum ambiguities into biological expression against the restraints imposed by the constant buffeting of an agitating external environment. Signals of all kinds, whether molecular or beyond, are information as energy. Each is derived from thermodynamic imperatives and both propel and compel biological results in reiterating levels of cellular entanglement. Biologic form emerges from that extension outward into the environment and back in a consistent reciprocation. Yet, the center of this decision matrix is always at the level of the self-referential cell whose identity is defined by a circumscribing Pervasive Information Field. As such, it is always both participant and enactor of further iterative environmental responses. All the mechanisms of communication that our research has identified sustain this perspective. Therefore, eukaryotic evolution is now understood as the means by which self-referential individual “principles of motion” collaborate through entanglement based upon information transfer whose communicative purpose is organized problem-solving. From this essential form of interchange, phenotype emerges as self-organizing cellular solutions in biologic form. If it is asserted that any good theory must be testable and falsifiable, then this definition becomes a direct research manifesto.

Evolution is decidedly an assertion of creativity that always dwells within both light and shade. Although creativity is certainly information based, it is unclear whether it skips along its interfaces or as phase transitions of contextual information. Yet in biologic terms, one aspect of information is necessarily true; it is both actualized information as physical state and concomitant ambiguity. Life is the dual faculty of using information and sensing its uncertainties and limitations, which in the same instantiation becomes its self-referential status. Those cellular actions that manifest as collective and emergent cellular solutions are achieved despite ambiguities towards the perpetual sustenance of a self-referential center at every scope and scale. Therefore, just as with our own thought processes that jostle within a realm of complex quantum entanglement, the discrete connections between those steps may always remain elusive. Yet, even within those necessary impediments, biology can now be better defined. In evolution, the past is ever prologue and is always a continuous enactment of quantum relativity, related to the subjective status of the observer/participant, and then settled. The process is perpetual. Quantum uncertainties are inherent to epiphenomena and flux against a bounded thermodynamically derived state function of “self”. The cusp of life is the ability to use information to sense ambiguities, and then settle them for better or worse. The ability to use information as another form of energy, and thereby actively discharge a range of implicates, defines life. In that sense, biology becomes metaphor and evolutionary development thereby reduces. At every scope and scale, it is the reiterative entangled property of living entities to use information to resolve environmental ambiguities into explicate self-referential biological solutions.
